# Cost-effectiveness of implantable cardioverter-defibrillators for primary prevention in heart failure with reduced ejection fraction: a Markov model using JROAD-DPC cost data in Japan

**DOI:** 10.3389/fcvm.2026.1744517

**Published:** 2026-03-05

**Authors:** Kazuki Ohashi, Masaya Watanabe, Yasuhiro Morii, Hisashi Yokoshiki, Kengo Kusano, Katsuhiko Imai, Masahiko Takagi, Teiichi Yamane, Hiroshi Tada, Katsuhiko Ogasawara

**Affiliations:** 1 Faculty of Health Sciences, Hokkaido University, Sapporo, Japan; 2 Department of Cardiovascular Medicine, Caress Memorial Hospital, Sapporo, Japan; 3 Department of Cardiovascular Medicine, Hokkaido University Graduate School of Medicine, Sapporo, Japan; 4 Center for Outcomes Research and Economic Evaluation for Health, National Institute of Public Health, Wako, Japan; 5 Department of Cardiovascular Medicine Sapporo City General Hospital, Sapporo, Japan; 6 Department of Cardiovascular Medicine, National Cerebral and Cardiovascular Center Hospital, Suita, Japan; 7 Department of Cardiovascular Surgery, Kure Medical Center, Kure, Japan; 8 Cardiovascular Surgery, Hiroshima University, Hiroshima, Japan; 9 Division of Cardiac Arrhythmia, Kansai Medical University Medical Centre, Moriguchi, Japan; 10 Department of Cardiology, The Jikei University School of Medicine, Tokyo, Japan; 11 Department of Cardiovascular Medicine, Faculty of Medical Sciences, University of Fukui, Fukui, Japan; 12 Faculty of Engineering, Muroran Institute of Technology, Muroran, Japan

**Keywords:** cost-effectiveness analysis, implantable cardioverter-defibrillator, left ventricular ejection fraction, primary prevention, sudden cardiac death

## Abstract

**Introduction:**

Implantable cardioverter-defibrillators (ICDs) reduce the risk of sudden cardiac death caused by ventricular tachycardia or ventricular fibrillation in patients with ischemic and non-ischemic cardiomyopathy. However, the cost-effectiveness of ICD implantation in Japanese patients with heart failure and reduced left ventricular ejection fraction remains unclear. This study aimed to evaluate the cost-effectiveness of ICD implantation in a Japanese setting.

**Methods:**

A Markov model with 1-month cycles was developed to assess the cost-effectiveness of ICD implantation compared with conventional medical therapy. The analysis was conducted from the perspective of a public healthcare payer over a 30-year time horizon. Scenario analyses accounting for waning treatment effects were performed, as along with deterministic and probabilistic sensitivity analyses (PSA).

**Results:**

In the base-case analysis, the incremental cost-effectiveness ratio (ICER) was US $29,838 per quality-adjusted life year (QALY). In the scenario analyses, the ICER increased to US $40,205 and $36,199 per QALY when the treatment effect began to wane after 5 and 10 years, respectively. ICD efficacy and battery longevity had the greatest influence on the ICER. PSA showed that the ICER per QALY ranged from US $19,472 at the 2.5th percentile to US $83,365 at the 97.5th percentile.

**Conclusions:**

In the Japanese healthcare context, ICD implantation for primary prevention was found to be more cost-effective than the reference value. However, depending on several assumptions, the ICER may exceed the reference value. Sensitivity analyses highlighted the significant impact of the hazard ratio and battery longevity on cost-effectiveness. Further research is needed to identify subpopulations with significantly different cost-effectiveness outcomes.

## 1 Introduction

Implantable cardioverter-defibrillators (ICDs) can reduce the risk of sudden cardiac death due to ventricular tachycardia or ventricular fibrillation in ischemic and non-ischemic cardiomyopathy (1). Clinical guidelines from Europe (2), the United States (3), and the Japanese Circulation Society/Japanese Heart Rhythm Society (4) recommend ICD implantation for primary prevention in patients with a left ventricular ejection fraction (LVEF) of 35% or less and heart failure symptoms classified as New York Heart Association (NYHA) class II or III, despite receiving adequate medical therapy. However, patients must manage various complications after ICD implantation, such as infection, lead failure, inappropriate shock, and battery replacement. Such treatment pathways affect patients’ quality of life and increase medical expenses.

Previous studies have performed cost-effectiveness analyses (CEA) of ICDs for the primary prevention of sudden cardiac death. Cowie et al. reported that prophylactic ICDs may be cost-effective in European healthcare settings. Their study showed incremental healthcare costs of €46,413 and a gain of 1.57 quality-adjusted life years (QALYs) (5). Holbrook et al. conducted a CEA of ICDs in a Taiwanese setting (6) and reported an incremental cost of NT $1,017,863 and a gain of 1.5 QALYs. Thus, they concluded that the incremental cost-effectiveness ratio (ICER) was NT $708,711 per QALY, which was below their predefined reference value (NT $2,100,000 per QALY), indicating that ICD implementation was cost-effective. The NICE reported that ICERs for ICD treatment ranged from £14,231 to £29,756 per QALY (7), concluding that ICDs could be considered cost-effective compared with optimal medical therapy if the maximum acceptable ICER was £30,000 per QALY. In summary, ICD implementation for the prevention of sudden cardiac death has been shown to be cost-effective in several countries. Although a CEA of ICD implementation for patients with Brugada syndrome (BrS) has been conducted in Japan (8), the cost-effectiveness of ICDs in patients with heart failure and reduced LVEF remains unclear. Furthermore, in Japan, ICD device and lead costs are reimbursed at approximately 3 million yen (9); however, recent improvements in ICD battery longevity have reduced the need for device replacement and associated treatment costs (10). Therefore, a CEA that considers technological innovations in the Japanese setting can provide useful information regarding the insurance system and clinical decision-making.

The estimated number of new ICD implantations in Japan is approximately 6,000 cases annually (11), representing a significant budgetary impact on the healthcare insurance system, especially considering the relatively high device costs. Therefore, the results of a CEA can support value-based decision-making. However, evidence regarding the context of CEA in Japan remains limited. As mentioned earlier, although several CEAs have been conducted overseas, they have not considered the differences in device costs or recent improvements in battery longevity. In addition, directly applying findings from overseas research to the Japanese context may lead to discrepancies due to differences in healthcare systems. Therefore, this study aimed to conduct a CEA of ICD implementation for the primary prevention of sudden cardiac death using real-world data from Japan, thereby addressing the limitations of previous studies and providing evidence applicable to clinical practice.

## 2 Methods

### 2.1 Target population

In this study, the clinical effectiveness and complications of ICD therapy were primarily sourced from previous studies, including the SCD-HeFT, whereas cost data and healthcare resource use were derived from the nationwide Japanese registry database, JROAD-DPC. The target population comprised patients with chronic heart failure classified as NYHA class II or III. All included patients had a reduced LVEF (≤35%) and a QRS duration <120 ms. The mean age of the population was 65.5 ± 11.6 years, and the majority were men (78.3%) (12). This population was selected based on the SCD-HeFT (13) and a Japanese cohort study (12). Comorbidities were ignored; however, their impact was included in the all-cause mortality outcomes.

### 2.2 Model settings

A Markov multistate model with a 1-month cycle was developed to conduct the CEA. The simulation was conducted over a 30-year time horizon. Our model compared ICDs [in addition to conventional medical therapy (CMT)] with CMT for the primary prevention of sudden cardiac death based on the SCD-HeFT study (13). The model consisted of three states: (i) stable; (ii) hospitalization, which included admissions for cardiac arrest (CA), heart failure, or complications; and (iii) death. Mortality was modeled using survival data from the pharmacotherapy arm of the SCD-HeFT long-term observational study (14). The survival curve was digitized and fitted using parametric survival models using Guyot's method (15). Among the candidate distributions, the log-logistic model was selected based on the Akaike Information Criterion and the Bayesian Information Criterion and was subsequently incorporated into the economic model. Patients remained hospitalized for one cycle before transitioning to either a stable or dead state. All patients who survived the initial ICD implantation were assumed to undergo device replacement every 10 years due to battery depletion. According to recent reports by manufacturers and clinical opinion, the expected battery longevity of this model is approximately 10 years (16, 17). Finally, the model was used to calculate the ICER based on the incremental costs and utilities associated with ICDs. The analysis was conducted from the perspective of a public healthcare payer (18).

### 2.3 Efficacy and safety of implantable cardioverter-defibrillator therapy

The hazard ratio (HR) for all-cause mortality associated with ICD implementation for primary prevention was 0.69 [95% confidence interval (CI): 0.55–0.87], as derived from a meta-analysis of previous randomized clinical trials (1). In the base-case analysis, we assumed that the treatment effects of ICDs would persist throughout the 30-year time horizon. In addition to reducing mortality, ICD therapy reduced hospitalizations due to CA and heart failure in this model. The annual probability of CA was 0.14% in the ICD group and 6.51% in the CMT group (19, 20). The annual probability of heart failure was 8% in the CMT group. Remote monitoring via ICDs reduced heart failure hospitalizations (incidence rate ratio (IRR) 0.75) (21, 22), which was attributable to the remote monitoring function of the device rather than its defibrillation ability. The annual incidences of hospitalization due to infection and lead failure during follow-up after ICD implantation were estimated to be 0.69% and 0.74%, respectively (23–25). The annual incidence of inappropriate shocks was 0.9% (26). The inputs are summarized in Table 1.

**Table 1:** Model input parameters.

Parameters (lower, upper)	ICDs	CMT	Distribution	Data source
Population				
Age	65.5 ± 11.6		Normal^a^	(12, 13)
Sex (male)	78.3%			
LVEF	≤35%			
NYHA	Ⅱ or Ⅲ			
Transition probability				
Hazard ratio for the probability of death	0.69 (0.55, 0.87)	—	Log-normal	(1, 14)
Probability of CA hospitalization	0.0014	0.0651 (0.043, 0.16)	Beta	(19, 20)
Probability of heart failure hospitalization	0.08			(21, 22)
Incident rate ratio for heart failure hospitalization	0.75 (0.45, 1.27)	—	Log-normal	
Probability of complications	0.0143 (0.007, 0.028)	—	Beta	(23–26), Assumption
Costs (US $)				
Initial intervention	30,550 (24,440, 36,660)		Gamma	JROAD-DPC
Device replacement	21,635 (17,308, 25,962)		Gamma	
CA hospitalization	4,918 (3,934, 5,902)		Gamma	
HF hospitalization	5,441 (4,353, 6,529)		Gamma	
Complications^b^	30,784 (24,627, 36,941)		Gamma	
Monthly follow-up cost^c^	34 (27, 41)		Gamma	
Utility				
Stable	0.74 (0.592, 0.888)		Beta	(7, 26, 27)
CA hospitalization	0.59 (0.472, 0.708)		Beta	
Heart failure hospitalization	0.68 (0.544, 0.816)		Beta	
Complications^b^	0.64 (0512, 0.768)		Beta	
Device replacement	−0.05	—	—	
Others				
Battery longevity (years)	10 (5, 12)	—	Triangular	(16, 17), Assumption
Discount rate (%)	2 (0, 4)		Beta	(18)

ICD, implantable cardioverter-defibrillator; CMT, conventional medical therapy; LVEF, left ventricular ejection fraction; NYHA, New York Heart Association; CA, cardiac arrest.

a If the value exceeded ±SD, the samples were resampled.

b Includes infection and lead failure.

c Includes remote monitoring and inappropriate shock.

### 2.4 Costs

We included the costs of the initial intervention, device replacement, hospitalization (i.e., cardiac arrest, heart failure, and complications), and additional follow-up (including remote monitoring and inappropriate shock). Costs were estimated from analyses of the JROAD-DPC database (approval number: 20240001) and the Japanese medical fee schedule, under which reimbursement is uniformly set for each medical service (9). The database is administered by the Japanese Circulation Society and includes Diagnosis Procedure Combination/Per-Diem Payment System data from approximately 1,500 hospitals as of 2024, covering the period from 2012 to 2024 (27, 28). This is one of the largest databases for cardiac and vascular diseases in Japan.

Using the JROAD-DPC database, we identified hospitalizations for the initial intervention and device replacement with or without complications (i.e., infection and lead failure as major complications) as well as hospitalizations for CA and heart failure based on the principal diagnosis and/or relevant procedures. Complication-related hospitalizations were extracted separately for each complication type according to prespecified definitions (Supplementary Table S1). For each admission, we obtained the all-inclusive total hospitalization costs recorded in the database. Following previous studies (23–25), we calculated the average per-complication hospitalization cost as a frequency-weighted mean of the total costs across complication types, using the observed incidence of each complication as weights. The monthly follow-up cost (including the costs of remote monitoring and inappropriate shocks) was set at US $34 based on the national fee schedule.

In Japan, ICD device costs, which account for a large part of the total cost, have decreased over the years. Because not using the most recent fees would likely affect the results, the utilization frequency of each device was extracted, the device costs were adjusted to the most recent values.

The inputs for the cost parameters are listed in Table 1. All costs were discounted at an annual rate of 2%, according to the guidelines (18).

### 2.5 Utility

QALYs were used as the primary outcome measurement in this analysis. Utility was based on the EuroQol five-dimension questionnaire from previous studies (7, 29, 30). As Japan-specific utility values were unavailable, we applied utility estimates from these published sources. The SHIFT study (30), which most closely resembled the population in the present study, was combined with the distribution of NYHA classes reported in the SCD-HeFT study to estimate QALY values using weighted averages, as follows: stable, 0.74; hospitalization (CA, 0.59; heart failure, 0.68; complications, 0.64); and death, 0. For device replacement, a disutility of −0.05 was applied (7) (Table 1). All utilities were discounted at an annual rate of 2%, according to the guidelines (18).

### 2.6 Scenario and sensitivity analyses

We conducted a scenario analysis to assess the uncertainty related to treatment-effect duration and the analytical time horizon. For treatment-effect waning (TEW), motivated by concerns noted in the NICE report (7), the treatment effect was assumed to decline over time following implementation (31): the waning began at 5 or 10 years, with the HR increasing linearly to 1.0 by year 20 and remaining at 1.0 thereafter. Given the limited clinical evidence regarding TEW, we aligned our TEW assumptions with the approach described in the NICE report (7). Separately, we also tested alternative time horizons (20 and 40 years) to assess structural uncertainty. These time-horizon scenarios did not apply treatment-effect waning.

In addition, deterministic sensitivity analysis (DSA) and probabilistic sensitivity analysis (PSA) were performed to evaluate the parameter uncertainty and its impact on the model outcomes. The ranges and distributions for each parameter were derived from previous studies (Table 1). For example, the base-case HR was 0.69, but the lower-effectiveness setting (HR: 0.87) reflected populations with complex complications or reduced ICD efficacy, as reported in the DANISH study (24). The cost and utility ranges were set at ±20%. PSA was performed using Monte Carlo simulation with 10,000 iterations to assess the parameter uncertainty, and results were presented using scatter plots. All analyses were performed using R version 4.4.1 (32). Costs were converted to US $ using the exchange rate as of 4 April 42025 (US $1 = JPY 146.26) (33).

### 2.7 Ethical statement

The research protocol was reviewed and approved by the Institutional Review Board of the Japanese Heart Rhythm Society (approval number: 20240001).

## 3 Results

### 3.1 Base-case and scenario analyses

In the base-case analysis, the per capita cost was US $61,119 for ICD therapy compared with US $7,626 for CMT; thus, the incremental cost of ICD therapy was US $53,492. ICD therapy provided 9.04 QALYs, whereas CMT provided 7.24 QALYs, resulting in an incremental gain of 1.79 QALYs. The ICER was US $29,838 per QALY (Table 2). In the scenario analysis, the ICER was US $40,205 per QALY when the treatment effect was assumed to wane after 5 years and US $36,199 per QALY when the effect was assumed to wane after 10 years (Table 2). The ICERs were US $36,738 per QALY and US $28,635 per QALY when the time horizon was set to 20 and 40 years, respectively.

**Table 2:** Cost, QALY, and ICER in base-case and scenario analyses.

Strategy	Total cost (US $)	Incremental cost (US $)	Total QALY	Incremental QALY	ICER (US $ per QALY)
Base-case analysis					
ICDs	61,119	53,492	9.04	1.79	29,838
CMT	7,626	—	7.24	—	—
Scenario analysis					
ICDs (5 years)^a^	59,241	51,614	8.53	1.28	40,205
ICDs (10 years)^b^	59,970	52,343	8.69	1.45	36,199

ICDs, implantable cardioverter-defibrillators; CMT, conventional medical therapy; QALY, quality-adjusted life year; ICER, incremental cost-effectiveness ratio.

a The treatment effect was assumed to last for 5 years.

b The treatment effect was assumed to last for 10 years.

### 3.2 Sensitivity analysis

**Figure 1:**
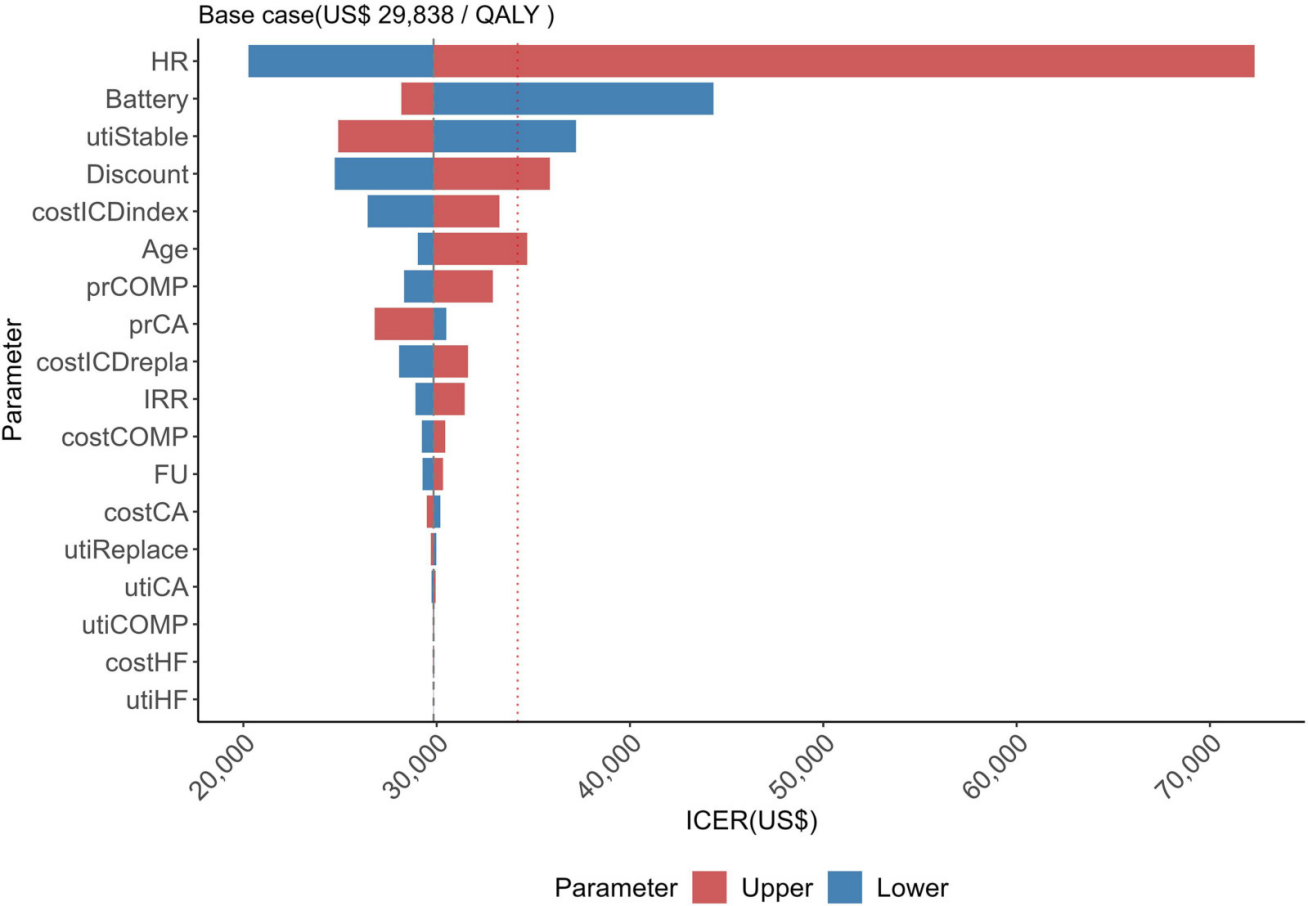
The range of ICER values obtained by varying each parameter between its upper and lower limits. The red dashed line represents the reference value of US$34,186 per QALY. HR, hazard ratio; Battery, battery longevity; utiStable, utility of stable state; Discount, discount rate; costICDindex, cost of initial intervention; prCOMP, probability of complications; prCA, probability of cardiac arrest; costICDrepla, cost of device replacement; IRR, incidence rate ratio; costCOMP, cost of complications; costFU, cost of follow-up; costCA, cost of cardiac arrest; utiReplace, utility of device replacement; utiCA, utility of cardiac arrest; costHF, cost of heart failure; utiCOMP, utility of complications; utiHF, utility of heart failure.

DSA identified ICD efficacy and battery longevity as the factors with the greatest impact on the ICER (Figure 1). The ICER ranged from US $20,263 to $72,311 based on changes in ICD efficacy and from US $28,173 to $44,318 based on changes in battery longevity.

**Figure 2:**
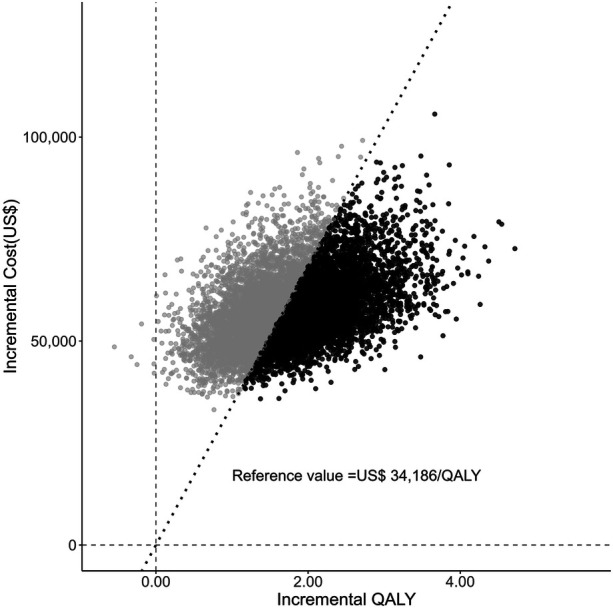
Each point represents the incremental cost and incremental quality-adjusted life years (QALYs) of ICD compared with the comparator, based on 10,000 Monte Carlo simulations. The median incremental cost-effectiveness ratio (ICER) was US $33,455 per QALY (2.5th-97.5th percentile: US$19,471-83,365). The diagonal dashed line represents the reference threshold of US $34,186 (JPY 5,000,000) per QALY. Points falling below this threshold indicate scenarios in which the incremental cost-effectiveness ratio is considered cost-effective and are shown as dark markers.

The PSA results are shown in Figure 2. The median ICER was US $33,455 per QALY, with a range from US $19,471 at the 2.5th percentile to US $83,365 at the 97.5th percentile. At a reference value of US $34,186 (JPY 5,000,000) per QALY, the probability that ICD was cost-effective was 52.7%.

## 4 Discussion

We conducted a cost-effectiveness analysis comparing ICD therapy with CMT to evaluate the cost-effectiveness of ICD implantation for primary prevention in Japanese patients with a reduced LVEF (≤35%). In the base-case analysis, the ICER was US $29,838 per QALY, which is below the Japanese reference value of US $34,186 per QALY for cost-effectiveness, indicating that ICD therapy is cost-effective (34). The key drivers with a significant impact on the results were ICD treatment efficacy and battery longevity.

Previous CEAs of ICDs for the primary prevention of sudden cardiac death have concluded that ICD therapy is cost-effective (5, 6, 35–37). The incremental QALY gain associated with ICD therapy in our results (1.79 QALY) was similar to those reported by Cowie et al. (1.57 QALY), Holbrook et al. (1.5 QALY), and Begisbayev et al. (1.51 QALY). Sun et al. conducted a CEA of ICDs for primary prevention of sudden cardiac death in China and reported an incremental QALY gain of 1.83 (37). Although we did not model cause-specific death due to a lack of evidence, our model was able to capture all-cause death from all states; therefore, the results remain comparable to those of previous results. However, while the ICER (US $29,838 per QALY) was acceptable in the Japanese setting, it was comparatively more cost-effective and acceptable in other countries. For example, a CEA of ICDs conducted in Taiwan reported an ICER of NT $0.7 million (US $21,537, NT $1 = US $0.03). Potential factors for this difference may include the cost and frequency of device replacement. Our initial intervention cost (US $30,550) was higher than that reported in Taiwan (NT $633,678 = US $19,010) (6). In addition, all surviving patients were assumed to undergo device replacement every 10 years. In summary, the differences from previous studies (6) were due to the higher initial costs and the model in which a greater number of individuals underwent battery replacements.

Scenario analyses considering treatment-effect waning (31) resulted in higher ICERs: US $40,205 per QALY when the treatment effect persisted for 5 years and then gradually declined to year 20, and US $36,199 per QALY when the effect persisted for 10 years with a gradual decline until year 20. These values exceeded the Japanese reference ICER value. There is no consensus on the assumptions about treatment-effect waning in health technology assessments (31). In the SCD-HeFT study, a comparison between the initial (13) and long-term outcomes (14) showed that the hazard ratio of ICD therapy increased from 0.76 to 0.87 over time, approaching 1.0. A more rapid decline in treatment effect or a shorter duration than assumed in this study would further reduce cost-effectiveness.

DSA revealed ICD efficacy and battery longevity as the key drivers in this analysis. These results are consistent with those of previous studies (5, 6, 37). These findings imply that targeting ICD therapy to patients most likely to derive substantial clinical benefit is essential and that healthcare systems may improve value for money by promoting devices with longer battery longevity. Large-scale non-inferiority randomized controlled trials evaluating the clinical benefit of ICDs in the current treatment era, such as the ongoing PROFID EHRA trial (38), which compares ICD therapy with contemporary optimal pharmacological management, suggest that the survival benefit of ICDs may be smaller under present therapeutic conditions. In our base-case model, we adopted a HR of 0.69 for ICD efficacy; however, if the incremental benefit of ICD therapy over modern drug therapy is attenuated, applying a more conservative HR of 0.87 may better reflect real-world clinical practice. Furthermore, it has been noted that older age at the time of ICD implantation is associated with poor cost-effectiveness (6, 39). However, the sensitivity analysis revealed that the effect of age was relatively small. This result can be attributed to the assumption that the treatment effect remains consistent across different age groups.

PSA assessed the uncertainty in the results for the expected parameter variations as US $19,471 to $83,365 per QALY (2.5th to 97.5th percentile). The cost-effectiveness of ICD therapy for the primary prevention of sudden cardiac death varies around the Japanese reference value. When combined with the DSA results, ICD efficacy had a significant impact. This suggests that ICD therapy is more likely to be cost-effective in patients with a lower risk of non-cardiac mortality (i.e., fewer comorbidities) and in populations in which a larger ICD treatment effect has been reported, such as patients with ischemic heart disease (1). Therefore, careful selection of treatment targets can lead to a cost-effective therapeutic approach, highlighting the need for future data accumulation to identify populations with favorable cost-effectiveness. Effective patient selection is emphasized as a key consideration in the Japanese clinical guidelines for arrhythmia treatment (4). The DANISH study (24) revealed no significant benefit of ICD therapy in patients aged 70 years or older. Given the limited life expectancy and the higher burden of comorbidities often observed in elderly patients (4), decisions regarding device replacement should be carefully individualized, with thorough discussion of the anticipated benefits and potential risks (40).

### 4.1 Limitations

Our study has several limitations. First, our model did not specify the causes of death, such as sudden cardiac death or heart failure, but simulated the impact of hospitalization costs by incorporating hospitalizations due to CA and heart failure. Second, our analysis was limited to ICD therapy and cannot be extrapolated to devices with cardiac resynchronization. We clarified the intended scope by restricting the target population to patients with QRS durations of <120 ms. However, some patients may be eligible for either ICD therapy or a cardiac resynchronization therapy defibrillator (CRT-D) in clinical practice. Accordingly, our results should be interpreted with caution when applied to such overlapping populations. If the model were expanded to incorporate CRT-D-specific costs and effects, it could be used to evaluate the cost-effectiveness of CRT-D as well. Finally, because this study was conducted within a Japanese setting, generalizing the results to other countries requires updating costs to reflect each healthcare system. Moreover, if data are available, country- or population-specific utility values and treatment-effect estimates should also be used.

## 5 Conclusion

This study conducted a CEA to compare ICD therapy with CMT for the primary prevention of sudden cardiac death in a Japanese setting from the perspective of a public healthcare payer. In the base-case analysis, the ICER for ICD therapy was US $29,838 per QALY. In the scenario analysis incorporating TEW, the ICER was estimated to be US $40,205 and $36,199 per QALY. Sensitivity analyses showed that ICD efficacy and battery longevity had substantial impacts on the results. Given that the ICER was close to commonly accepted cost-effectiveness reference values in Japan, ICD therapy has the potential to be a cost-effective treatment option, particularly when targeted to appropriately selected patient populations.

## Data Availability

The data analyzed in this study are subject to the following licenses/restrictions: the deidentified participant data will not be shared. Requests to access these datasets should be directed to https://www.j-circ.or.jp/jittai_chosa/.
