# Supplementary material for: Cost-effectiveness of implantable cardioverter-defibrillators for primary prevention in heart failure with reduced ejection fraction: a Markov model using JROAD-DPC cost data in Japan
Source: Front Cardiovasc Med. 2026 Mar 5;13:1744517. doi: 10.3389/fcvm.2026.1744517 (PMC12999398; doi:10.3389/fcvm.2026.1744517)
Supplement: Supplementary file 1 [file Table1.docx]

Supplementary

Table S1 showed the definitions used to extract cost data from the JROAD-DPC database.

|  | Definitions | |
| --- | --- | --- |
| Items | ICD-10/SCD/MPC/MSC | Surgical procedure code |
| Initial Intervention | None | K599 |
| Device replacement | None | K599-2 |
| Hospitalization | A41.0, A41.1, A41.8, A41.9, I319, I400, I810, T140, T814, T793, T827-3,  I971&QP92(SCD),  J048(MPC),  733370000(MSC) | K920 |

Table S1

ICD-10, International Classification of Diseases-10; SCD, Standardized Disease Code; MPC, Medical Procedure Code; MSC, Medical Supply Code
